# Deep Gluteal Syndrome and Persistent Sciatica in a Patient With Unilateral Piriformis Muscle Hypertrophy: A Case Report

**DOI:** 10.1002/ccr3.73730

**Published:** 2026-10-12

**Authors:** Najoua Ghani

**Affiliations:** ^1^ Faculty of Medicine Euromed University of Fez Fez Morocco

**Keywords:** case report, deep gluteal syndrome, image‐guided injection, musculoskeletal ultrasonography, piriformis muscle, sciatica

## Abstract

A man of 47 had 6 months of left sciatica without root compression on lumbar imaging. Pelvic CT showed isolated left piriformis hypertrophy, 700 versus 450 mm^2^. Guided betamethasone injection relieved him within 48 h, sustained at 6 months. Asymmetry and response support, but do not prove, sciatic nerve entrapment.

## Key Clinical Message

Deep gluteal syndrome should be considered when sciatica is persistent and poorly systematized despite non‐contributory lumbar imaging. Piriformis hypertrophy on pelvic imaging and relief after an image‐guided injection support the working diagnosis and guide effective conservative management, but neither establishes mechanical entrapment of the sciatic nerve; ultrasonography now allows bilateral morphometric, enthesal and dynamic assessment that cross‐sectional imaging alone does not provide.

## 1 Introduction

Sciatica is a symptom rather than a diagnosis, and only part of it arises from lumbar nerve‐root compression. Deep gluteal syndrome (DGS) designates non‐discogenic disorders of the sciatic nerve caused by entrapment within the deep gluteal space, and has been defined in a systematic review by three elements: A non‐discogenic disorder, involvement of the sciatic nerve, and entrapment within the deep gluteal space [1].

The concept has broadened substantially over the past 5 years and now extends well beyond the historical notion of piriformis syndrome. Contemporary reviews emphasize that structures other than the piriformis muscle belly may generate deep gluteal pain: Enthesopathy of the piriformis and of the other deep external rotators, muscle–tendon pathology acting as a primary pain generator, calcific lesions of the piriformis tendon or of the sacrospinous ligament, ischiofemoral impingement, fibrovascular bands and variant sciatic nerve anatomy [2, 3, 4]. Within this framework, a hypertrophic piriformis is one possible contributor among several rather than a diagnosis in itself.

Historically, piriformis syndrome was estimated to account for approximately 6% of cases of sciatica [5]. Prevalence estimates remain unreliable because diagnostic criteria are not uniform and because clinical features overlap extensively with those of lumbosciatica [6].

Diagnostic strategy has changed in parallel. Conventional computed tomography (CT) and magnetic resonance imaging (MRI) mainly serve to exclude competing causes, while magnetic resonance neurography, diffusion tensor imaging, provocative electrophysiology and quantitative muscle morphometry have been proposed as more specific tools, with modest diagnostic performance [7, 8]. High‐resolution ultrasonography has emerged as the only widely available modality that combines bilateral morphometric comparison, direct visualization of the sciatic nerve, assessment of the piriformis enthesis and of calcific deposits, dynamic evaluation of nerve gliding during hip rotation, and real‐time guidance of injection without ionizing radiation [3, 4, 9, 10, 11].

We report a case of DGS associated with unilateral piriformis hypertrophy that responded completely to a CT‐guided corticosteroid injection. Our purpose is not to claim proof of mechanical entrapment, but to set out explicitly the diagnostic reasoning that supported the working diagnosis, the alternative causes that were excluded, and the limits imposed by an imaging pathway that included neither MRI nor ultrasonography nor electrodiagnostic testing.

### 1.1 Anatomical Background

The piriformis arises from the anterolateral surface of the sacrum, lateral to the second to fourth sacral foramina, passes through the greater sciatic foramen and inserts on the apex of the greater trochanter, dividing the foramen into a suprapiriform compartment, traversed by the superior gluteal nerve and vessels, and an infrapiriform compartment, traversed by the sciatic nerve, the posterior femoral cutaneous nerve, the pudendal nerve and the inferior gluteal pedicle. The deep gluteal space is bounded posteriorly by the gluteus maximus, anteriorly by the posterior acetabular column and the hip capsule, medially by the sacrotuberous and sacrospinous ligaments, laterally by the linea aspera, superiorly by the inferior border of the gluteus medius and inferiorly by the proximal hamstring origin [1, 2].

## 2 Case Presentation

### 2.1 Patient Information

A 47 year‐old man, a long‐distance lorry driver, was referred to the rheumatology department for a supervised exercise reconditioning programme for chronic low back pain. He reported a one‐year history of intermittent exertional low back pain rated 30–50/100 on a visual analogue scale (VAS). Over the preceding 6 months he had developed poorly systematized left‐sided sciatica terminating at the knee, of progressively increasing intensity rated 60–70/100, Treatment with a paracetamol–caffeine combination, piroxicam and thiocolchicoside was ineffective, and rehabilitation had been discontinued because of pain. There was no history of gluteal trauma, intramuscular gluteal injection, hip or pelvic surgery, no fever, weight loss or inflammatory night pain, and no sphincter or perineal symptoms. The clinical course is summarized in Figure 1.

**FIGURE 1:**
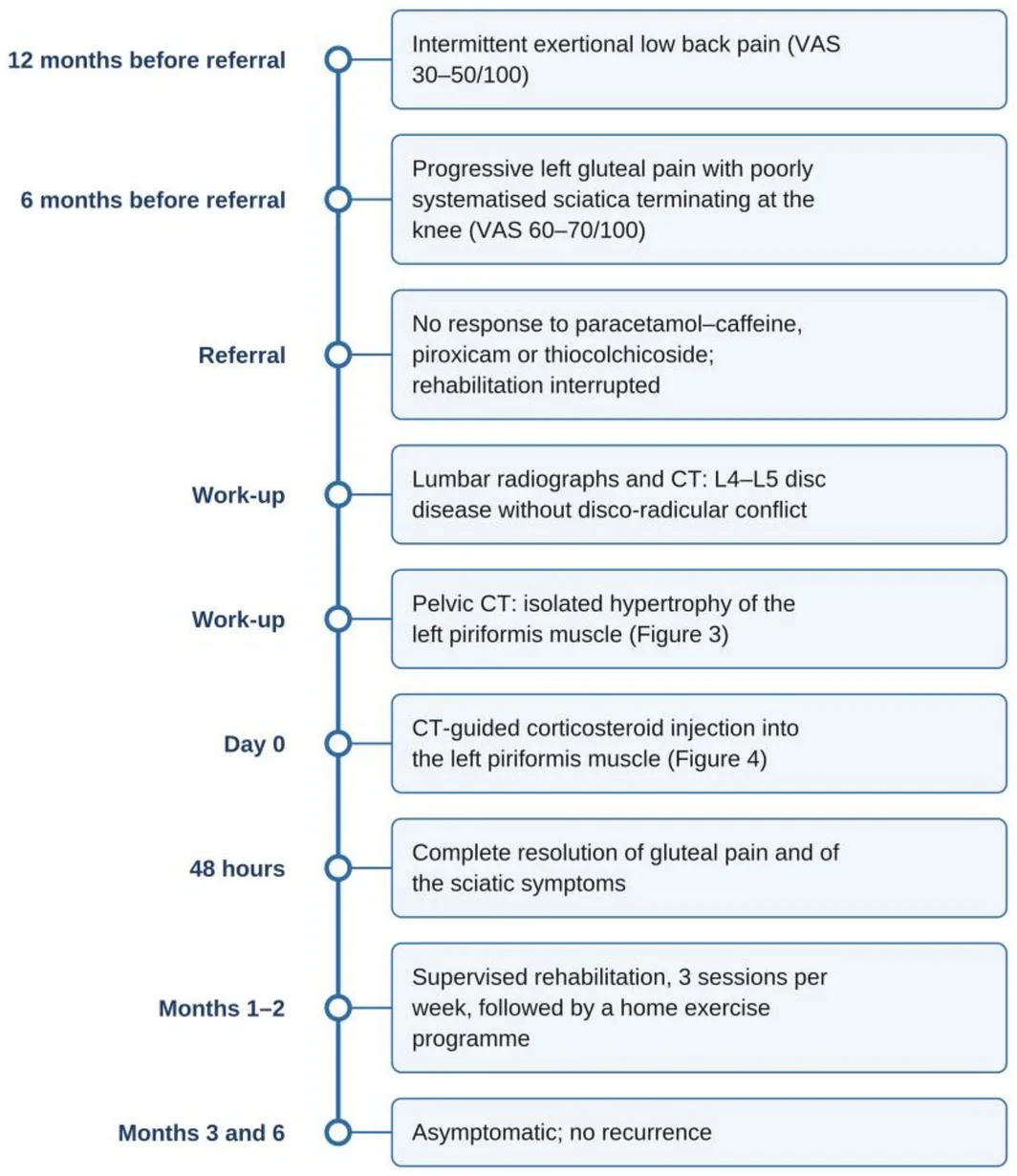
Clinical timeline, from the onset of exertional low back pain to the six‐month follow‐up. VAS, visual analogue scale; CT, computed tomography.

### 2.2 Clinical Findings

General examination was unremarkable; body mass index was 23 kg/m^2^. Examination of the lumbar spine showed stiffness, with a Schober index of 3.5 cm; there were no inflammatory features of back pain and no peripheral synovitis, enthesitis, dactylitis or extra‐articular manifestation suggesting spondyloarthritis.

Neurological examination of both lower limbs was normal: muscle strength was 5/5 in all L2–S1 myotomes, there was no sensory deficit in the L4, L5 or S1 dermatomes, patellar and Achilles reflexes were present and symmetrical, plantar responses were flexor, and there was no saddle hypoaesthesia or sphincter disturbance.

All of the provocative tests specific to DGS were performed in this patient, bilaterally and comparatively (Figure 2):

- deep palpation of the buttock posterior to the greater trochanter, midway between the sacrum and the greater trochanter, reproduced the patient's usual pain on the left and was painless on the right;
- the Pace and Nagle test (seated resisted abduction) was positive on the left and negative on the right (Figure 2a);
- the flexion–adduction–internal rotation (FAIR) test was positive on the left and negative on the right (Figure 2b);
- the Beatty maneuver was positive on the left and negative on the right (Figure 2c);
- the seated piriformis stretch test and the active piriformis test were both positive on the left and negative on the right;
- the Freiberg maneuver was positive on the left and negative on the right;
- the straight‐leg raise was negative bilaterally, both with and without internal rotation of the limb.

**FIGURE 2:**
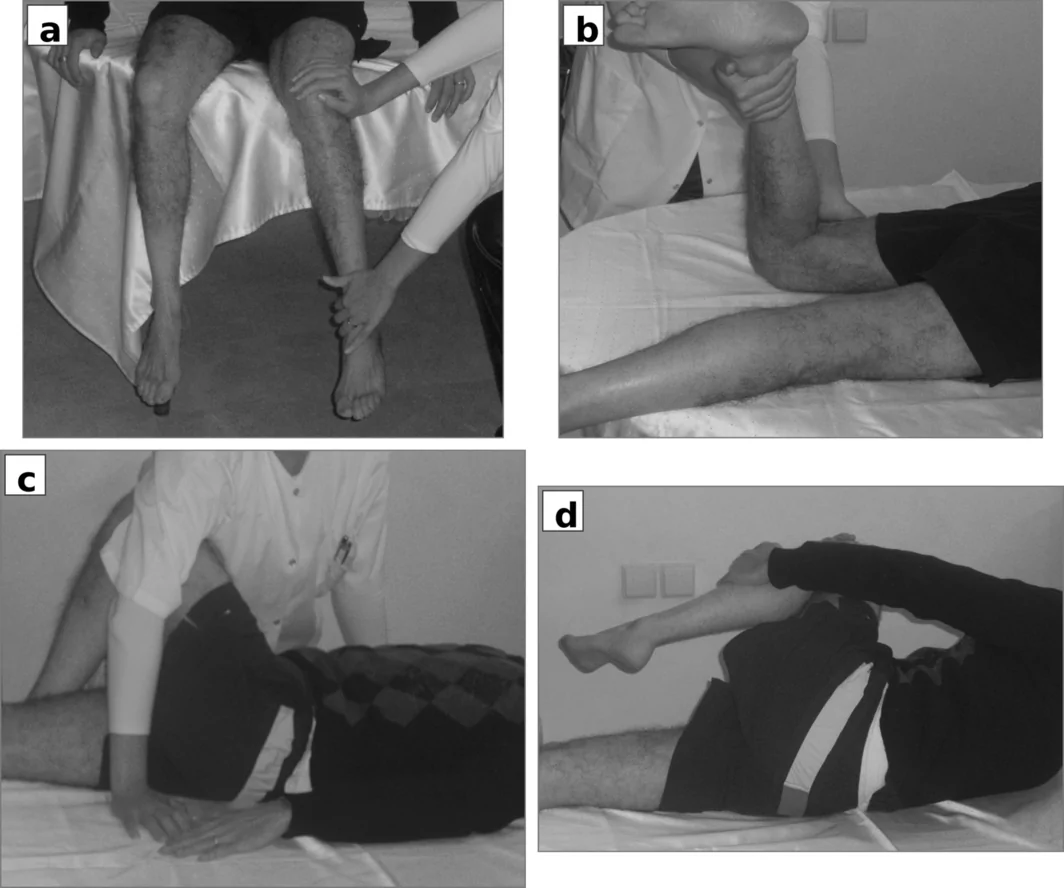
Provocative maneuvers of the deep gluteal space, photographed in the present patient with his written informed consent. (a) Pace and Nagle test: Seated resisted abduction. (b) Flexion–adduction–internal rotation (FAIR) test in the supine position. (c) Beatty maneuver in lateral decubitus on the asymptomatic side. (d) Self‐administered stretching of the piriformis in flexion, adduction and internal rotation. The three provocative maneuvers illustrated were all performed in this patient and were all positive on the symptomatic side.

Competing sources of deep gluteal pain were assessed clinically: there was no tenderness over the ischial tuberosity or the proximal hamstring origin, none over the lateral facet of the greater trochanter, and none over the sacroiliac joints; sacroiliac provocation tests were negative; and hip range of motion was complete and painless in all planes.

### 2.3 Diagnostic Assessment

Inflammatory markers were normal: C‐reactive protein 6 mg/L and erythrocyte sedimentation rate 13 mm in the first hour.

Plain radiographs of the lumbar spine showed L4–L5 disc disease. Lumbar CT confirmed L4–L5 disc disease without disco‐radicular conflict, with no foraminal or lateral recess stenosis and no lumbosacral transitional anomaly.

Pelvic CT demonstrated hypertrophy of the left piriformis muscle (Figure 3). Because the report rests on this asymmetry, the muscle was measured retrospectively on the original dataset. On the axial slice showing the largest muscle area, the cross‐sectional area of the piriformis was 700 mm^2^ on the left and 450 mm^2^ on the right, a side‐to‐side difference of 250 mm^2^ and a left‐to‐right ratio of 1.56. Measurements were obtained independently by a radiologist and a rheumatologist, and the values reported are the consensus reading. For comparison, a piriformis cross‐sectional area above 611.67 mm^2^ on hip MRI has been proposed as a threshold for piriformis syndrome, with an area under the curve of 0.81 and a sensitivity and specificity of 75% [12]. In the present patient, the left piriformis lay above that threshold and the right below it. This comparison should nevertheless be read with caution: the threshold was derived on MRI, its transposition to CT has not been validated, and its discriminative performance is moderate.

**FIGURE 3:**
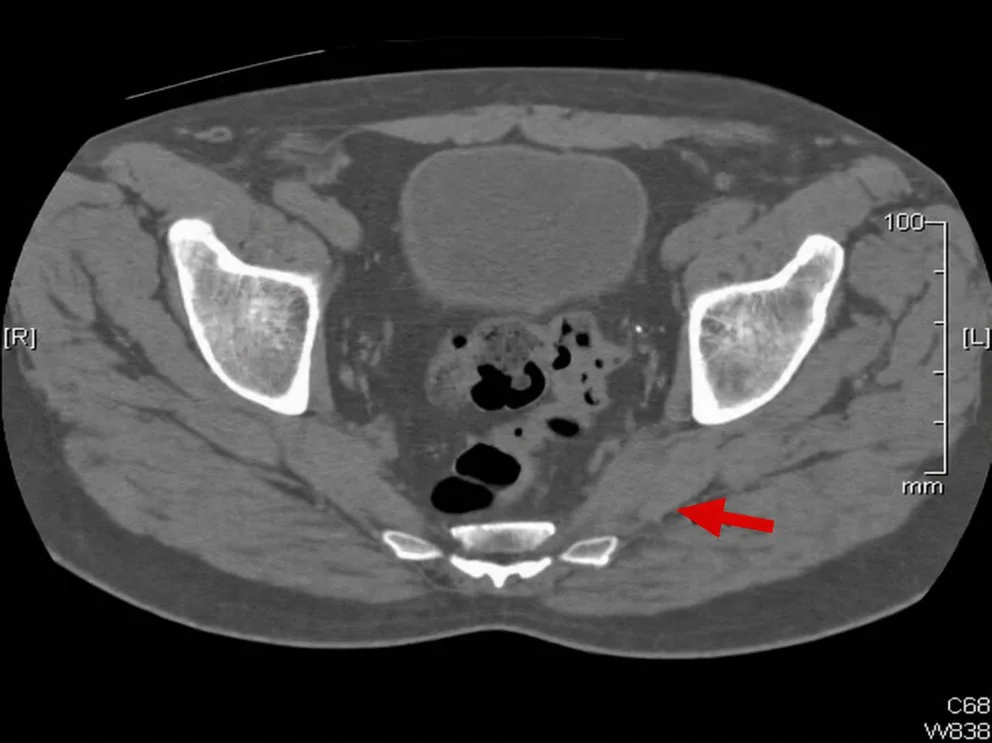
Axial pelvic computed tomography showing hypertrophy of the left piriformis muscle (arrow). The bilateral cross‐sectional areas measured on the original dataset are reported in Section 2.3.

The same examination showed no alternative cause of deep gluteal pain: no calcification of the piriformis tendon, of the sacrospinous ligament or of the hamstring origin; no ischiofemoral narrowing; no gluteal or pelvic mass; no bone lesion; no sacroiliitis; and normal hip joints. The sciatic nerve itself was not separately resolved on CT. No MRI, magnetic resonance neurography, ultrasonography or electrodiagnostic study was performed.

### 2.4 Therapeutic Intervention

With the patient prone, a CT‐guided injection into the left piriformis muscle was performed through a posterior transgluteal approach (Figure 4). A 22‐gauge, 90‐mm spinal needle was advanced under intermittent CT control until the tip lay within the muscle belly. No iodinated contrast medium was used. A total volume of 4 mL was then injected: 1 mL of a betamethasone dipropionate and betamethasone sodium phosphate suspension (Diprostène; betamethasone dipropionate 6.43 mg, equivalent to 5 mg of betamethasone, and betamethasone sodium phosphate 2.63 mg, equivalent to 2 mg of betamethasone, i.e., a total of 7 mg of betamethasone per mL), together with 3 mL of 2% lidocaine (60 mg). The procedure was well tolerated, with no immediate or delayed complication.

**FIGURE 4:**
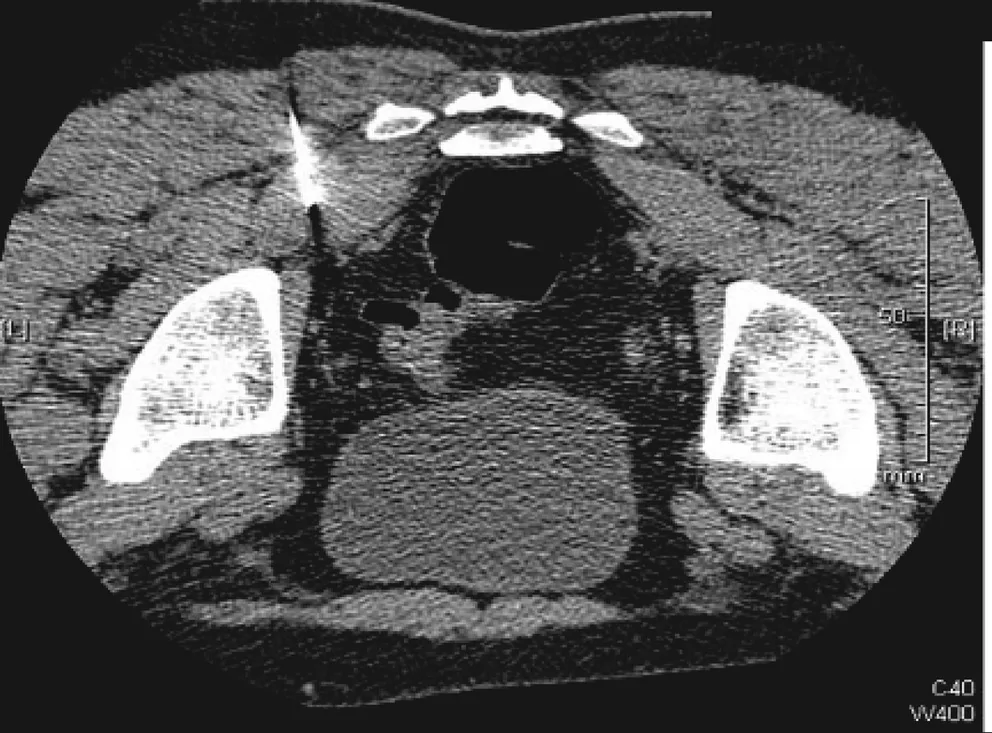
Computed tomography‐guided injection into the left piriformis muscle: Patient prone, posterior transgluteal approach, with the needle in place in the left deep gluteal space.

### 2.5 Follow‐Up and Outcomes

Gluteal pain and the sciatic symptoms resolved completely within 48 h, with the VAS falling from 60 to 70/100 before the procedure to 0/100. Rehabilitation was resumed for 2 months at three sessions per week. This was the same standard exercise reconditioning programme for chronic low back pain that had been interrupted before the injection; no piriformis‐specific physiotherapy was prescribed. It was followed by a self‐directed exercise programme. At three and 6 months the patient was completely asymptomatic, with a pain score of 0/100. No validated disability score was collected prospectively, and no adverse event was reported.

## 3 Discussion

### 3.1 Diagnostic Reasoning in This Patient, and Its Limits

Several elements converge in this patient: persistent sciatica poorly systematized to a single root; absence of disco‐radicular conflict on lumbar CT; focal tenderness within the deep gluteal space; reproduction of the usual pain by every piriformis‐specific provocative maneuver on the symptomatic side, each negative contralaterally, with a negative straight‐leg raise; unilateral piriformis hypertrophy; absence of an alternative cause on pelvic CT; complete relief within 48 h of a targeted injection; and sustained remission at 6 months.

Convergence is not proof, and anatomical asymmetry alone does not establish symptomatic entrapment of the sciatic nerve. Morphometric thresholds discriminate only moderately (an area under the curve of 0.81 with sensitivity and specificity of 75% for cross‐sectional area [7, 12]), and the sciatic nerve passes through or splits around the piriformis in approximately 17% of individuals, so neither the presence nor the absence of asymmetry is decisive [7, 13]. In this patient we obtained neither MRI nor magnetic resonance neurography, neither electrodiagnostic testing including the H‐reflex in the FAIR position nor any direct assessment of the nerve. The negative straight‐leg raise is compatible with a deep gluteal origin but has no confirmatory value, and no single provocative maneuver is diagnostic: the best‐performing combination, the active piriformis test with the seated piriformis stretch test, reached a sensitivity of 0.91 and a specificity of 0.80 against endoscopic findings, in a small and selected surgical population [14]. Both tests were positive in this patient, which strengthens the clinical argument without removing that limitation.

We therefore regard the diagnosis in this patient as a probable, clinically and radiologically coherent DGS in which piriformis hypertrophy is the most plausible contributing factor and not as a demonstrated mechanical entrapment of the sciatic nerve by the piriformis muscle.

### 3.2 The Contemporary Concept of Deep Gluteal Syndrome

Two mechanisms have classically been proposed for piriformis‐related sciatic pain: friction of the muscle against the underlying neural structures, irritating the sciatic trunk and the posterior femoral cutaneous nerve [15], and compression of these structures between the piriformis and the ischium [16]. They are not mutually exclusive. Recent work has shifted attention from the muscle belly to the whole musculotendinous unit: enthesopathy of the piriformis and of the other deep external rotators may act as a primary pain generator, with or without nerve involvement [2, 4], and calcification of the piriformis tendon or of the sacrospinous ligament has been described as a cause of sciatica that conventional imaging may under‐recognize and that ultrasonography identifies readily [3]. Other recognized contributors include ischiofemoral impingement, quadratus femoris oedema, fibrovascular bands, gluteal tendinopathy, proximal hamstring pathology, and variant nerve anatomy [1, 7, 8, 13]. This is directly relevant to the present case: we can state that pelvic CT showed neither calcification nor ischiofemoral narrowing, but we cannot exclude an enthesopathic component at the trochanteric insertion, which CT resolves poorly and which was not assessed dynamically.

### 3.3 Imaging Strategy: Why Computed Tomography Here, and What Ultrasonography Would Have Added

CT was chosen for pragmatic rather than conceptual reasons: no interventional ultrasound facility was available in our centre at the time of management, and the interventional radiology team was experienced in CT‐guided procedures. It allowed the lumbar spine and the pelvis to be assessed in the same session, excluded bone and space‐occupying lesions, and provided a reproducible route for the guided injection.

CT was nonetheless the weakest link in this work‐up. It does not reliably resolve the sciatic nerve, provides no information on nerve gliding, characterizes the piriformis enthesis poorly, and delivers ionizing radiation to the pelvis.

Ultrasonography would have addressed most of these limitations. It permits side‐to‐side comparison of piriformis thickness with good interrater reliability (intraclass correlation coefficient 0.836) [11] and against a published diagnostic threshold [10]; direct measurement of sciatic nerve caliber and echotexture at the site of entrapment, with comparison to the asymptomatic side [9]; assessment of the piriformis enthesis and detection of calcific deposits [3, 4]; dynamic evaluation during passive and active hip rotation, which is the only practical way to demonstrate impaired nerve gliding or motion‐related changes at the enthesis [4, 9]; and real‐time guidance of injection or of sciatic nerve hydrodissection without ionizing radiation [17]. Its limitations are equally real: operator dependence, the depth of the deep gluteal space in obese patients, and a restricted field of view for pelvic alternatives such as intrapelvic masses. We therefore regard ultrasonography and cross‐sectional imaging as complementary rather than competing. In retrospect, an ultrasonographic examination with dynamic assessment before the procedure, and ultrasound rather than CT guidance for the injection itself, would have strengthened this report substantially, and this is the sequence we would adopt today.

### 3.4 Interpreting the Response to Injection

The complete and durable response is clinically important but of limited diagnostic value. Injected corticosteroid diffuses beyond its target and may relieve pain arising from the enthesis, from the neighboring external rotators or from peri‐neural tissue as readily as pain arising from a compressed nerve; no anesthetic‐only control block was performed. A randomized trial found improvement after ultrasound‐guided local anesthetic injection without an additional benefit from corticosteroid [18], whereas a later systematic review and meta‐analysis reported pain reduction with local anesthetic, corticosteroid, their combination and botulinum toxin, while emphasizing heterogeneous designs, small samples and limited generalisability [19]. Spontaneous improvement and the resumption of rehabilitation may also have contributed, although the programme resumed was a general reconditioning programme for chronic low back pain that included no piriformis‐specific physiotherapy, which limits that particular confounder. A favorable response therefore supports the working diagnosis and justifies the therapeutic strategy; it does not demonstrate that a hypertrophic piriformis mechanically entrapped the sciatic nerve.

### 3.5 Management Options

First‐line management is conservative: activity modification, analgesia, correction of biomechanical factors, and rehabilitation combining contract–relax work and stretching in hip flexion, adduction and internal rotation. When symptoms persist, image‐guided injection of local anesthetic, corticosteroid or botulinum toxin may be considered [18, 19]. Evidence for botulinum toxin remains of fair quality for pain reduction but insufficient to establish the magnitude of benefit, functional outcomes or an optimal dose [20]; in a retrospective cohort, CT‐guided injections containing botulinum toxin were associated with a higher early response rate and a longer response duration than injections without it [21]. Ultrasound‐guided sciatic nerve hydrodissection and prolotherapy have been reported as alternatives targeting the nerve–muscle interface and the enthesis respectively [3, 4, 17]. A systematic review of conservative and surgical treatments judged the overall quality of evidence to be low and could not recommend one conservative treatment over another [22]. Endoscopic sciatic nerve decompression is reserved for carefully selected patients with refractory symptoms and objective evidence of entrapment; a recent systematic review of 312 patients reported success rates of 70%–100%, with a 2.5% recurrence rate and a 1.6% revision rate [23].

### 3.6 Strengths and Limitations

The strengths of this report are the concordance between the clinical findings, the exclusion of disco‐radicular conflict and of alternative pelvic causes on imaging, the demonstration of unilateral piriformis hypertrophy, the rapid improvement after an anatomically targeted intervention, and the sustained resolution at 6 months, together with a documented chronology, pain intensity, injectate, rehabilitation schedule and follow‐up.

The limitations are substantial and we state them explicitly. First, the diagnosis rests on a clinical and morphological convergence rather than on direct evidence of nerve involvement: no MRI, magnetic resonance neurography, ultrasonography or electrodiagnostic testing was performed. Second, only part of the battery of provocative tests described for DGS was applied. Third, the therapeutic response cannot be attributed with certainty to the corticosteroid or to relief of a mechanical conflict. Fourth, no validated disability score was recorded, so the functional outcome is described qualitatively. Fifth, follow‐up is limited to 6 months, whereas recurrence in DGS may occur later. Finally, all quantitative imaging data reported here were obtained retrospectively from the original examination; no datum has been reconstructed from memory, and any item not recoverable from the medical record has been reported as unavailable.

## 4 Conclusion

Deep gluteal syndrome (DGS) is an important cause of non‐discogenic sciatica. Piriformis muscle hypertrophy may contribute to sciatic nerve involvement within the deep gluteal space, but imaging asymmetry is not by itself diagnostic. In the present case, unilateral piriformis hypertrophy was demonstrated by pelvic CT after lumbar imaging had excluded disco‐radicular conflict, and CT‐guided injection of betamethasone (total dose 7 mg) was followed by complete pain resolution within 48 h, resumption of rehabilitation and sustained clinical remission at 6 months. This observation supports considering DGS when persistent sciatica is not explained by lumbar nerve‐root compression, and illustrates the value of an anatomically targeted injection. It also illustrates the limits of a CT‐only pathway: in current practice, ultrasonography, with bilateral morphometric comparison, assessment of the enthesis and dynamic evaluation of the sciatic nerve, should be integrated early, and the diagnosis should be formulated as a probable rather than a proven mechanism.

## Author Contributions

**Najoua Ghani:** conceptualization, investigation, funding acquisition, writing – original draft, methodology, validation, visualization, writing – review and editing, software, formal analysis, project administration, data curation, supervision, resources.

## Funding

The author has nothing to report.

## Disclosure

Figure Originality, Sources and Permissions: Every remaining figure originates from the present case. Figure 1 is a timeline created by the author. Figure 2a–d are clinical photographs of the patient taken during the index examination; they contain no identifying feature and are published with the patient's written informed consent. Figures 3 and 4 are the patient's own CT images, obtained at Mohammed V Hospital, Meknes, Morocco, and published with the same written consent. No figure has been reproduced or adapted from a third party, and no permission from a third‐party rights holder is therefore required.

## Ethics Statement

Ethics committee approval was not required for a single case report at our institution.

## Consent

Written informed consent was obtained from the patient for the publication of this case report and of the accompanying clinical and radiological images. The signed consent form has been retained and can be provided confidentially on request.

## Conflicts of Interest

The author declares no conflicts of interest.

## Data Availability

The data that support the findings of this study are available on request from the corresponding author. The data are not publicly available due to privacy or ethical restrictions.
